# Evaluation of Antibiotic Supply Decisions by Community Pharmacists in Thailand: A Vignette Study

**DOI:** 10.3390/antibiotics10020154

**Published:** 2021-02-03

**Authors:** Sisira Donsamak, Marjorie C. Weiss, Dai N. John

**Affiliations:** 1 School of Pharmacy and Pharmaceutical Sciences, Cardiff University, Cardiff CF10 3NB, UK; WeissM1@cardiff.ac.uk; 2 Faculty of Pharmaceutical Sciences, Ubon Ratchathani University, Warin Chamrab, Ubon Ratchathani 34190, Thailand

**Keywords:** community pharmacist, pharmacy, survey, antibiotic, Thailand

## Abstract

In Thailand, antibiotics are available lawfully from community pharmacies without a prescription. Inappropriate supply of antibiotics from Thai community pharmacies to the public for common, self-limiting diseases has been reported. The study aimed to evaluate the appropriateness of antibiotics selected by community pharmacists in Thailand in response to vignettes. A cross-sectional survey of community pharmacists across Thailand was conducted using a self-administered questionnaire including nine case vignettes with three conditions, namely upper respiratory infections (URIs), acute diarrhoea and simple wounds. A total of 208 questionnaires were completed and analysed (20.8% response rate). In response to vignettes relating to URIs, 50.8% of pharmacist recommendations were not in accordance with antibiotic guidelines. Inappropriate recommendations for diarrhoea and wound cases were 20.8% and 16.7%, respectively. A higher proportion of younger pharmacists, those with less experience, Pharm. D. graduate pharmacists, employee pharmacists and those pharmacists who worked in a chain pharmacy were more likely to recommend appropriate antibiotic treatment in response to the vignettes (*p* < 0.05). These findings will be useful to promote educational interventions for community pharmacists regarding common infectious disease management in order to improve appropriate antibiotic use.

## 1. Introduction

In recent years, the problem of antimicrobial resistance (AMR) has increased significantly and has become a serious health care issue worldwide [1,2,3,4]. Growing resistance to antibiotics is a particularly serious global challenge. AMR-related infections are estimated to contribute to approximately 700,000 deaths per year, globally. Failing to tackle AMR could cause 10 million deaths a year and cost up to USD 100 trillion by 2050 [4]. Inappropriate use of antibiotics accelerates antibiotic resistance [2,5,6,7].

Community pharmacists are easily accessible to the public in many countries [8]. Most antibiotic consumption occurs in the community, and antibiotics are obtained from community pharmacies with or without a prescription. The inappropriate supply of antibiotics from community pharmacies has been attributed to several factors, for example, lack of knowledge of pharmacists and pharmacy staff, demand from customers, financial incentives and a lack of regulation and/or enforcement of existing regulations [1,9,10,11,12,13].

Antibiotic supplies without a prescription from community pharmacies have been found to have contributed to the inappropriate use of antibiotics, particularly in developing countries, even though antibiotic supply without a prescription is prohibited by the law [14,15,16,17,18]. In Thailand, antibiotics are widely available lawfully from community pharmacists without the need for a prescription. Antibiotic supplies without a prescription from community pharmacies have been found to have contributed to the inappropriate, including over-use, of antibiotics in Thailand [19]. Several studies in Thailand showed that over fifty per cent of patients who visited a community pharmacy with upper respiratory infections (URIs) or acute diarrhoea were supplied with antibiotics [20,21,22,23].

In Thailand, the Antibiotics Smart Use (ASU) Program was implemented in 2007. This programme aimed to reduce unnecessary use of antibiotics for common self-limiting conditions, including URIs, acute diarrhoea and simple wounds [24]. In 2017, the Thailand national strategic plan on antimicrobial resistance (2017–2021) was established to promote multisectoral collaboration in order to reduce antimicrobial consumption, to reduce AMR morbidity and to raise public awareness [25].

Thai practice guidelines for the management of URIs, acute diarrhoea and simple wounds in community pharmacies have been published [26,27]. According to the practice guidelines, antibiotics are not recommended for most patients who present with one of these three conditions. In addition, the use of antibiotics for non-bacterial infections will pose a risk of adverse drug reactions and contribute to the development of antibiotic resistance. The supply of antibiotics from community pharmacies to the public for common, self-limiting conditions such as some URIs, acute diarrhoea and simple wounds is common and often does not comply with the guidelines [22,28,29]. Others have used surveys of healthcare professionals, which have included vignettes to identify intentions as a proxy for their practice [30,31,32]. Therefore, this study was conducted to evaluate the appropriateness, according to Thai guidelines, of the intended supply of antibiotics for URIs, acute diarrhoea and simple wounds by community pharmacists in Thailand using vignettes. The study also aimed to determine the association between the demographic data of community pharmacists and the appropriateness of the intended supply, or otherwise, of antibiotics.

## 2. Results

### 2.1. Demographics of Respondents

A total of 211 community pharmacists out of the thousand pharmacists in the sample responded to the survey. Ninety questionnaires were returned by post; two questionnaires were excluded because of the return of a blank questionnaire. One hundred twenty-one respondents in the sample completed the questionnaire. The response rate was 20.8%. Demographic data of respondents is shown in Table 1.

### 2.2. Recommending Antibiotics Based on Vignettes (Intention to Supply)

About half (49.2%, 306 out of 622 instances) of treatment recommendations for the three URI vignettes (case a, b, and c in Table 2) were inappropriate according to the Thai guidelines [26,27]. On the other hand, 20.8% (*N* = 130/624) and 16.7% (*N* = 104/622) of antibiotic recommendations for the three diarrhoea cases (d, e and f) and three wound vignettes (g, h and i), respectively, were inappropriate (Table 2).

Even though the pharmacists were correct (according to the guidelines) in recommending an antibiotic for the vignette cases, some of them suggested inappropriate antibiotic regimens.

For vignette “a”, a child who was unlikely to have a Group A Streptococcal (GAS) infection, antibiotics were not recommended by the practice guidelines. However, 48.1% (*N* = 100/208) of community pharmacists incorrectly recommended antibiotics for this vignette. The most commonly suggested antibiotic was amoxicillin (91.0%, *N* = 91/100). Other suggested antibiotics were azithromycin (2.0%, *N* = 2/100), roxithromycin (1.9%, *N* = 2/108), co-amoxiclav (1.0%, *N* = 1/100), clarithromycin (1.0%, *N* = 1/100) and erythromycin (1.0%, l, *N* = 1/100).

In total, 35.9% (*N* = 74/206) of respondents suggested a teenager, vignette “b”, to have antibiotics, while antibiotics might likely be of benefit. Of 74 respondents who suggested that an antibiotic was required, 19 (25.7%) selected an antibiotic treatment as recommended by the practice guidelines, that is, amoxicillin 500 mg twice daily for ten days. Other suggested antibiotics not in accordance with guidelines were roxithromycin (5.4%, *N* = 4/74), azithromycin (1.4%, *N* = 1/74), co-amoxiclav (1.4%, *N* = 1/74), and co-trimoxazole (1.4%, *N* = 1/74).

In the third URI vignette (vignette “c”), antibiotic treatment would be likely to be of benefit for the patient. In this case, 99.0% (*N* = 206/208) of pharmacists recommended antibiotics for the patient. However, only 22.8% (*N* = 47/206) of community pharmacists suggested appropriate antibiotic treatment as recommended by the practice guidelines. Amoxicillin was the most common antibiotic suggestion by respondents. However, 67 pharmacists (32.5%) stated they would supply too high a dose of amoxicillin, 1500–2000 mg per day. Almost half of participants (46.1%, *N* = 95/206) recommended a sub-optimal duration of amoxicillin treatment, mostly five to seven days instead of the recommended 10 days. The most common inappropriate antibiotic recommended was co-amoxiclav (22.8%, *N* = 47/206). Table 3 presents those antibiotics that were selected for vignettes where antibiotics are recommended by the Thai guidelines (vignette c, e and i).

Antibiotics were not required for case “d”, diarrhoea, with 97.6% of respondents (*N* = 208) not recommending antibiotics. Antibiotics recommended inappropriately were norfloxacin (80.0%, *N* = 4/5) and tetracycline (10.0%, *N* = 1/5).

The child in case “f” was likely to have had a viral infection, based on Thai guidelines, resulting in diarrhoea, for which antibiotics were not required. In total, 23.1% of pharmacists (*N* = 48/208) recommended antibiotics for this patient, which were nifuroxazide (29.2%, *N* = 14/48), co-trimoxazole (20.8%, *N* = 10/48), norfloxacin (15.1%, *N* = 8/48), azithromycin (10.4%, *N* = 5/48), amoxicillin (6.3%, *N* = 3/48), furazolidone (4.2%, *N* = 2/48), cefixime (4.2%, *N* = 2/48) and cefdinir (2.1%, *N* = 1/48). The other four pharmacists did not specify the name of the antibiotic.

For case “e”, the patient was likely to have shigellosis. About ninety per cent of pharmacists (90.9%, *N* = 189/208) recommended antibiotic treatment for this patient. Most of them (71.4%, *N* = 135/189) recommended an appropriate antibiotic treatment for shigellosis, ciprofloxacin 500 mg twice daily for 3 days (2.1%, *N* = 4/189) or norfloxacin 400 mg twice daily for 3-5 days (69.3%, *N* = 131/189). In terms of inappropriate recommendations, supplying antibiotics for longer than the recommended duration of treatment was the most common reason (12.7%, *N* = 24/189). Four respondents would refer the patient to consult a doctor.

Twenty-eight respondents (13.5%, *N* = 207) recommended antibiotic treatment for case “g” where antibiotics were not required. Antibiotics suggested for this case included dicloxacillin (67.9%, *N* = 19/28), cloxacillin (7.1%, *N* = 2/28), topical fusidic acid (6.7%, *N* = 2/28), topical gentamicin (6.7%, N = 2/28) and topical mupirocin (3.6%, *N* = 1/28).

For case “h”, 87.4% of participants (*N* = 181/207) did not recommend antibiotic treatment for the girl, which was the appropriate response. Antibiotics suggested for this case were dicloxacillin (34.6%, *N* = 9/26), cloxacillin (23.1%, *N* = 6/26), cephalexin (7.7%, *N* = 2/26), amoxicillin (3.8%, *N* = 1/26), co-amoxiclav (3.8%, *N* = 1/26, topical gentamicin 7.7%, (*N* = 2/26) and topical fusidic acid (3.8%, *N* = 1/26),

Almost all pharmacists (97.6%, *N* = 203/208) recommended antibiotics for the infected wound scenario “i”. Most pharmacists (77.8%, *N* = 158/203) suggested the correct antibiotic treatment as dicloxacillin 250–500 mg four times daily (74.9%, *N* = 152/203) or cloxacillin 500 mg four times daily (3.0%, *N* = 6/203).

### 2.3. Factors Influencing the Intention to Supply Antibiotics

According to the responses to the vignettes, appropriate recommendation scores were calculated (see Section 4.) The results are shown in Table 4. The maximum score for each condition was 3, in which the pharmacist had three correct responses for the three vignettes for that condition. The appropriate recommendation score for URI vignettes was low compared to the appropriate recommendation score for antibiotics for the diarrhoea and wound vignettes. Forty per cent (43.2%, *N* = 89/206) scored 1/3 points on the appropriate recommendation score of URI vignettes. Most pharmacists recommended appropriate antibiotic treatment for the diarrhoea and wound vignettes. About half of participants (52.2%, *N* = 108/207) scored 3/3 points on the appropriate recommendation score for diarrhoea vignettes. In addition, 64.6% (*N* = 133/206) of participants scored 3/3 points on the appropriate recommendation score for wound vignettes.

Bivariate correlations were undertaken to identify the demographic data that may be correlated with the intention-to-supply score for antibiotic treatment. The results are presented in Table 5. It can be seen that age and length of experience were found to be significantly correlated with the appropriate recommendation score for antibiotic supply. The findings showed that the appropriate recommendation scores were higher (that is, more appropriate supply) when the pharmacists’ age or length of experience was lower. Gender was found to be correlated with the appropriate recommendation score of antibiotic supply for wound vignettes (*p* = 0.01) and total recommendation score with no correlation found with other conditions. The education level of pharmacists was found to correlate with the total appropriate recommendation score. Pharmacists who graduated from a Pharm D programme were found to have a higher appropriate recommendation score than those who graduated with a BPharm and postgraduate degree (*p* < 0.05). The role of the pharmacist and type of community pharmacy were significantly correlated with appropriate recommendation score on antibiotic supply for URI vignettes, diarrhoea vignettes and total appropriate recommendation score. Pharmacists who were an employee or worked in a chain pharmacy were seen to have higher appropriate recommendation scores for URI vignettes, diarrhoea vignettes and for the total appropriate recommendation score. An accredited pharmacy was found to be correlated with the appropriate recommendation score of antibiotic supply for URIs vignettes (*p* = 0.04.)

## 3. Discussion

Nine vignettes, comprising three URI vignettes, three diarrhoea vignettes and three wound vignettes, were used to evaluate the appropriateness of the recommended antibiotics by community pharmacists. Appropriate recommendations for URI vignettes were poor as in over half of URIs instances (50.8%), inappropriate antibiotic treatments were recommended. Less than one quarter of antibiotic recommendations for the diarrhoea (20.8%) and wound (16.7%) vignettes were inappropriate. URIs are mostly self-limiting and normally no antibiotic treatment is needed, and most guidelines do not routinely recommend antibiotic treatments for many acute URIs [26,27,33,34]. Despite this, inappropriate antibiotic prescribing and supply for respiratory indications is widely reported. A high proportion of inappropriate supply of antibiotics for URIs has recently been reported in some developing countries. In China, a simulated client (SMC) study revealed that pharmacists supplied a high proportion of cases with antibiotics, 88.4% (130/147 cases) for acute cough [34]. Another SMC study of 2411 pharmacies in China [35] also reported 70.1% (*N* = 1690) of pharmacies supplied antibiotics for acute adult URIs. In addition, a study in Sri Lanka reported that 43.3% (26/60 pharmacies) of pharmacy staff supplied antibiotics for acute sore throat [36]. A study in Egypt showed that 98.3% (234/238) of pharmacy visits resulted in the supply of antibiotics for viral URIs cases [37]. Most of these studies showed a higher rate of inappropriate supply of antibiotics for URIs compared to what was found in the present study.

The inappropriately selected antibiotic treatments for the case vignettes in this study, across URIs, diarrhoea and simple wounds, may result from a lack of up-to-date knowledge regarding patient assessment and/or antibiotic treatments. A cross-sectional survey study in Shiraz, Iran [38] reported that 60.3% of 90 pharmacists had poor knowledge regarding the application of medicines used for the treatment of children’s diarrhoea. The authors also stated that the inability of pharmacists to completely assess the patient’s problem and the inaccurate diagnosis of the patients’ condition could lead to inappropriate recommendations. In addition, a cross-sectional survey study [39] in 703 community pharmacists in Southern Thailand concluded that pharmacists who were knowledgeable on the criteria used for GAS infection diagnosis were more likely to appropriately diagnose streptococcal pharyngitis and less likely to supply antibiotics inappropriately. Thus, more education regarding patient assessment and the antibiotic treatments for infectious diseases is needed to improve the rational supply of antibiotics from community pharmacists in Thailand.

In this study, the findings showed that age and length of experience in community pharmacy correlated with the intention to supply appropriate antibiotics. Higher proportions of younger pharmacists and pharmacists with less experience in community pharmacy stated an appropriate antibiotic. A similar association was also reported in a previous study: it was reported that greater practice experience in community pharmacy potentially increased the likelihood of inappropriate antibiotic use in Southern Thailand [40]. Likewise, a study in Lebanon found that pharmacists with more experience in pharmacies had less knowledge about the appropriate use of antibiotics compared to those with less experience [40]. Age and community pharmacy experience were positively correlated, as expected. Older age and longer practice experience possibly indicate that they had been qualified for a longer time; they might not keep up to date and/or their education might not have covered antibiotic use and AMR to the same level as more recent graduates.

The highest education level of pharmacists was also shown to be associated with the appropriateness of antibiotic supply. Pharmacists who graduated from a PharmD programme intended to supply antibiotics more appropriately than those who graduated with a BPharm. This is possibly the result of pharmacy education programme reforms in Thailand, transitioning from a 5-year bachelor’s degree programme to a 6-year Pharm D programme, which comes with enhanced clinical practice in the sixth year of study [41,42]. In addition, since 2016, almost all qualified pharmacists have a Pharm D degree.

The findings also showed that pharmacy owners were more likely to state that they would supply inappropriate antibiotics, as were community pharmacists who worked in independent pharmacies. Similarly, a cross-sectional survey study conducted in Bangkok and Chonburi in Thailand in 2017 found that pharmacists who worked in a chain pharmacy have more knowledge regarding antibiotic use than others who worked in an independent pharmacy [43]. This is possibly because employee pharmacists were usually recently graduated pharmacists. In line with this is a cross-sectional survey with 90 community pharmacists in Iran [38], which found that recently graduated pharmacists had more knowledge on medicines used for the treatment of diarrhoea than the ones who had graduated much earlier. Again, these findings are likely to support the idea that up to date knowledge towards antibiotic treatments is an essential factor for the appropriate supply of antibiotics from community pharmacists.

The Community Pharmacy Accreditation Project in Thailand was introduced in 2002 to ensure the delivery of high-quality pharmaceutical care by community pharmacies in Thailand. Surprisingly, accreditation status of pharmacy was found not to be significantly associated with the appropriate supply of antibiotics. Similar results were found in a mixed-methods study using observation followed by semi-structured interview in thirty community pharmacies in Vietnam. They found that there was no significant difference between Good Pharmacy Practice (GPP) certified pharmacies and non-GPP certified pharmacies regarding antibiotic supply practice [44]. The findings in the present study may be due to the fact that Thai pharmacy accreditation rules focus on the infrastructure of pharmacies. In terms of rational pharmacy practice, there are no checks to monitor and control the appropriateness of antibiotic supply practice. Therefore, the monitoring of the rational supply of medicines from community pharmacies may be needed to promote the appropriate supply of medicines in community pharmacies in Thailand.

Our findings revealed that many community pharmacists may lack up-to-date knowledge towards antibiotic use and antimicrobial susceptibility in Thailand. Therefore, education and training of community pharmacists regarding infectious disease management is needed. The WHO suggested establishing AMR as a core component of professional education, training, certification and development for the health sectors, including community pharmacies [45].

This is the first cross-sectional survey about antibiotic supply from community pharmacists in Thailand recruited across Thailand. There are three key limitations to the findings of this study. Firstly, the findings may not represent the wider practice and views of community pharmacists in Thailand. However, systematic random sampling was used to recruit community pharmacists for the postal survey. The study sample was diverse in terms of gender, age, length of experience, type of pharmacy and accreditation status of pharmacy and was from different regions of the country. Secondly, as this was a self-administered questionnaire-based study, there is the possibility that participants may have over-reported desirable practices or views or under-reported undesirable practices or views. As a result, the appropriateness of antibiotic supply by community pharmacists found in this study may represent a more favourable picture than might actually be the situation. Thirdly, the study asked about the intention to supply, rather than measuring actual supply.

## 4. Materials and Methods

### 4.1. Study Design

A cross-sectional survey using a self-completed questionnaire with Thai community pharmacists was used in this study. The questionnaires were distributed to selected community pharmacies by post. The data were collected between October and December 2019.

Stratified random sampling was conducted to recruit community pharmacies from all regions, each with different cultural, socio-economic and socio-demographic characteristics. To classify strata, firstly, community pharmacies were grouped based on their location into the four regions, namely central Thailand, Northern Thailand, Northeastern Thailand and Southern Thailand. Then, community pharmacies in each regional area were divided into three groups based on the population size of the province in which the pharmacy was located. Quota sampling was also used in order to recruit a reasonable number of accredited pharmacies to the study. A ratio of approximately 1:2 accredited pharmacies:non-accredited pharmacies was used (See Table S1).

### 4.2. Case Vignettes

Participants’ recommended antibiotic treatments were established through the use of vignettes. Nine vignettes, comprising three URI vignettes, three diarrhoea vignettes and three wound vignettes were included. Case vignettes were created based on a review of the literature and of Thai antibiotic practice guidelines [26,27]. The questionnaire and vignettes were sent for feedback to one academic pharmacist, two clinical pharmacists and two community pharmacists for face and content validity. The questionnaire was pilot-tested with fourteen community pharmacists.

Survey participants were asked if they would recommend antibiotics when the patient or caregiver was to present at their pharmacy with the specified symptoms and the person presenting did not ask for a specific medicine. The pharmacist participants were asked to indicate the name of the antibiotic they would recommend, dosage regimen and duration of treatment, or other option, including no supply. For each vignette, pharmacists were asked to consider that the person was able to afford the cost of medicines, and in each case, the person with symptoms had no comorbidity or undiagnosed underlying disease, used no other medication and had no history of drug allergy or intolerance. The appropriateness of antibiotic treatment indicated by community pharmacists was assessed based on the Thai practice guidelines for community pharmacists [26,27].

### 4.3. Appropriate Recommendation Score Calculation

The appropriate recommendation scores were calculated according to the appropriateness of recommended antibiotic treatments as intended by community pharmacists. The scores were used as a dependent variable to identify factors influencing the appropriateness of antibiotic supply. The correct answers were identified according to the Rational Drug Use in community pharmacy, Thailand guideline [26]. Correct answers, according to the guideline, were given a numerical value of “1”. On the other hand, “0” was given for incorrect answers as shown in Table 6.

### 4.4. Statistical Analysis

Data analyses were performed using IBM SPSS statistics 25. As the study consisted of two different methods of response using the same questionnaire, there were two data sets based on the survey (the postal survey and the open online survey). The homogeneity of variance and the difference between the demographic data of two datasets were tested to see if the survey data from the two methods could be combined. Kruskal–Wallis H test was used to determine if there were statistically significant differences between the two methods of responding to the survey with regards to the variance of community pharmacists’ appropriate recommendation score of antibiotic supply. Then, a chi-square test (for categorical variables) or a Mann–Whitney U test (for other variables) was used to test the difference between the two groups by looking at the demographic data.

Nonparametric tests were used to compare the data from both surveys due to the appropriate recommendation score not being normally distributed. Bivariate analyses were conducted to identify the factors that correlated with pharmacists’ appropriate recommendation score using Pearson’s correlation (for continuous variables) or Spearman’ rank test (for categorical variables).

### 4.5. Ethical Approval

Ethical approval was obtained from Cardiff University School of Pharmacy and Pharmaceutical Sciences Ethics Committee (in English 1819-22) and the Research Ethics Committee of Ubon Ratchathani University, Thailand (in Thai UBU-REC-28/2562).

## 5. Conclusions

Factors such as age, length of experience in community pharmacy, highest education level and employment status were found to be associated with the appropriateness of antibiotic supply. These findings may relate to a lack of up-to-date knowledge by community pharmacists regarding treatment and diagnosis of infectious disease.

## Figures and Tables

**Table 1:** Demographics of respondents.

Categories	Postal Survey, N (%) (*N* = 208)
**Age (years)**	
Median (IQR)	33 (29.0–41.8)
Minimum	24
Maximum	81
**Experience as community pharmacist (years)**	
Median (IQR)	6.0 (3.0–11.0)
Minimum	0.3
Maximum	44.0
Missing	1
**Gender**	
Male	64 (30.8)
Female	144 (69.2)
**Highest education**	
Bachelor’s degree in Pharmacy	119 (57.2)
Doctor of Pharmacy (Pharm D.)	67 (32.2)
Post-graduation degree	22 (10.6)
**Role in a pharmacy**	
Owner	115 (55.3)
Employee	93 (44.7)
**Type of pharmacy**	
Independent pharmacy	133 (63.9)
Chain pharmacy	74 (35.6)
Missing	1 (0.5)
**Accreditation status ^1^**	
No	88 (42.3)
Yes	120 (57.7)
**Region**	
Central	92 (44.2)
Northern	39 (18.8)
Northeastern	30 (14.4)
Southern	40 (19.2)
Missing	7 (3.4)

^1^ Community pharmacy accreditation in Thailand is voluntary. The accreditation criteria comprise five domains: premises and facilities, personnel, drug inventory and stock records, dispensing and patient care, and patient satisfaction and health promotion.

**Table 2:** Recommendation to supply antibiotics based on vignettes.

Vignettes	No ^1^ N (%)	Yes ^2^ N (%)	Missing
**URI**			
a. 6-year-old boy, weight 20 kg, presenting with a sore throat for 2 days accompanied by mild fever, productive cough with thick and coloured discharge. There are no other symptoms ^3^.	108 (51.9)	100 (48.1)	-
b. 14-year-old girl presenting with sore throat for 2 days, accompanied by high grade fever, no cough, no runny nose or any other symptoms. She is not pregnant or breast-feeding ^4^.	132 (63.5)	74 (35.6)	2 (1.0)
c. 43-year-old man with a severe sore throat for 2 days accompanied by high grade fever, tender lymph nodes, pus on tonsils but no cough. There are no other symptoms.	2 (1.0)	206 (99.0)	-
**Acute diarrhoea**			
d. 70 year-old-woman with watery stool 3 times within the last 12 h, no fever and no other symptoms. There are no signs of dehydration ^3^.	203 (97.6)	5 (2.4)	-
e. 30 year-old-woman with diarrhoea with blood visible in stools since yesterday evening, accompanied with high grade fever, and abdominal cramps. She is not pregnant or breast-feeding and has are no other symptoms.	19 (9.1)	189 (90.9)	-
f. 3 year-old-boy, weight 15 kg, with watery stool 4 times within the last 10 h accompanied by mild fever, nausea and mild abdominal pain. There is no sign of dehydration and there are no other symptoms ^3^.	160 (76.9)	48 (23.1)	-
**Simple wound**			
g. 35 year-old-man who had a motorcycle accident (about 15 min earlier) with many minor, superficial scratches on the left arm and left leg ^3^.	179 (86.1)	28 (13.5)	1 (0.5)
h. 7-year-old-girl who has a fresh, thin, shallow cut wound on left index finger about 1 cm long, which happened about 30 min earlier ^3^.	181 (87.0)	26 (12.5)	1 (0.5)
i. 50-year-old man who has a shallow wound on the right calf, about 1 cm in diameter. He had a cut wound by barbed wire about 4 days ago. The skin surrounding the wound has become red, swollen and sore, and with pus. The patient confirmed that he had a recent tetanus vaccination booster.	5 (2.4)	203 (97.6)	-

^1^ Pharmacist did not recommend antibiotics for case vignette. ^2^ Pharmacist recommended antibiotics for case vignette. ^3^ Antibiotics were not recommended according to the guidelines. ^4^ Antibiotics may be likely to be of benefit to the patient. Consider no antibiotic with advice or antibiotic treatment is based on pharmacist discretion.

**Table 3:** Appropriateness of antibiotic treatment recommended by community pharmacists.

Inappropriateness of Supplying Antibiotics	Recommended Antibiotic Treatment	Number (%)
***Group A streptococcal (GAS) infections case, case “c” (N = 206)***		
**Inappropriate drug choice**	co-amoxiclav	47 (22.8)
	dicloxacillin	1 (0.5)
	cephalexin	1 (0.5)
	azithromycin	6 (2.9)
	roxithromycin	2 (1.0)
	clarithromycin	1 (0.5)
	co-trimoxazole	1 (0.5)
**Too low a dose**	amoxicillin less than 1000 mg per day	1 (0.5)
**Too high a dose**	amoxicillin more than 1000 mg per day	67 (32.5)
**Inappropriate dosing interval**	amoxicillin three or four times daily	66 (32.0)
**Inappropriate duration**	amoxicillin treatment time less than 10 days	95 (46.1)
***Shigellosis case, case “e” (N = 189)***		
**Inappropriate drug choice**	metronidazole	16 (8.5)
	ofloxacin	4 (2.1)
	cefixime	1 (0.5)
	cefdinir	1 (0.5)
**Too low a dose**	ciprofloxacin 250 mg twice daily	1 (0.5)
**Too high a dose**	norfloxacin 800 mg twice daily	1 (0.5)
**Inappropriate duration of treatment**	ciprofloxacin for 5–10 days	11 (5.8)
	norfloxacin for 7–10 days	12 (6.3)
	norfloxacin less than 3 days	1 (0.5)
***Superficial skin infection wound, case “i” (N = 203)***		
**Inappropriate drug choice**	amoxicillin	2 (1.0)
	ampicillin	1 (0.5)
	co-amoxiclav	11 (5.4)
	cephalexin	3 (1.5)
	clindamycin	2 (1.0)
	metronidazole	1 (0.5)
**Improper dosing interval**	cloxacillin two or three times daily	321.2)
	dicloxacillin three times daily	10 (4.9)

**Table 4:** Appropriate recommendation score for community pharmacists in relation to antibiotics.

Appropriate Recommendation Score	
**Appropriate recommendation score for URI vignettes (*N* = 206)**	
Median (IQR)	1.0 (1.0–2.0)
Minimum–Maximum	0.0–3.0
**Appropriate recommendation score for diarrhoea vignettes (*N* = 207)**	
Median (IQR)	3.0 (2.0–3.0)
Minimum–Maximum	0.0–3.0
**Appropriate recommendation score for wound vignettes (*N* = 206)**	
Median (IQR)	3.0 (2.0–3.0)
Minimum–Maximum	0.0–3.0
**Total appropriate recommendation score (*N* = 204)**	
Median (IQR)	7.0 (5.0–7.8)
Minimum–Maximum	1.0–9.0

**Table 5:** Bivariate correlation between demographic data and appropriate recommendation score on antibiotic supply among community pharmacists.

	Appropriate Recommendation Score			
	URIs	Diarrhoea	Wound	Total
**Age ^1^**				
Correlation	−0.22	−0.24	−0.20	−0.33
*p*-value	<0.01	<0.01	<0.01	<0.01
**Length of experience ^1^**				
Correlation	−0.14	−0.16	−0.20	−0.24
*p*-value	<0.01	<0.01	0.01	<0.01
**Gender ^2^**				
correlation	0.11	0.10	0.21	0.19
*p*-value	0.11	0.16	<0.01	<0.01
**Education ^2^**				
Correlation	0.10	0.10	0.13	−0.16
*p*-value	0.17	0.06	0.07	0.03
**Role of pharmacist ^2^**				
Correlation	−0.17	−0.15	0.12	−0.19
*p*-value	0.02	0.04	0.09	<0.01
**Type of pharmacy ^2^**				
Correlation	0.17	0.15	0.12	0.19
*p*-value	0.02	0.04	0.09	<0.01
**Accreditation status ^2^**				
Correlation	0.14	0.09	0.05	0.05
*p*-value	0.04	0.19	0.52	0.61
**Regions ^2^**				
Correlation	0.22	0.13	0.01	0.09
*p*-value	0.70	0.06	0.91	0.22

^1^ Pearson’s correlation test, ^2^ Spearman’ rank test.

**Table 6:** Scoring for each vignette to assess the appropriateness of the antibiotic recommendation.

Vignettes	Antibiotics	Points
a. 6-year-old boy, weight 20 kg, presenting with a sore throat for 2 days accompanied by mild fever, productive cough with thick and coloured discharge. There are no other symptoms.	No	1
	Yes	0
b. 14-year-old girl presenting with sore throat for 2 days, accompanied by high grade fever, no cough, no runny nose or any other symptoms. She is not pregnant or breast-feeding and has are no other symptoms ^1^	No	1
	amoxicillin 500 mg twice daily for 10 days	1
c. 43-year-old man with a severe sore throat for 2 days accompanied by high grade fever, tender lymph nodes, pus on tonsils but no cough. There are no other symptoms	No	0
	amoxicillin 500 mg twice daily for 10 days	1
d. 70-year-old-woman with watery stool 3 times within the last 12 h, no fever and no other symptoms. There are no signs of dehydration.	No	1
	Yes	0
e. 30-year-old-woman with diarrhoea with blood visible in stools since yesterday evening, accompanied with high grade fever, and abdominal cramps. She is not pregnant or breast-feeding and has are no other symptoms.	No	0
	norfloxacin 400 mg twice daily for 3–5 days, or ciprofloxacin 500 mg twice daily for 3 days.	1
f. 3-year-old-boy, weight 15 kg, with watery stool 4 times within the last 10 h accompanied by mild fever, nausea and mild abdominal pain. There is no sign of dehydration and there are no other symptoms.	No	1
	Yes	0
g. 35-year-old-man who had a motorcycle accident (about 15 min earlier) with many minor, superficial scratches on the left arm and left leg.	No	1
	Yes	0
h. 7-year-old-girl who has a fresh, thin, shallow cut wound on left index finger about 1 cm long, which happened about 30 min earlier.	No	1
	Yes	0
i. 50-year-old man who has a shallow wound on the right calf, about 1 cm in diameter. He had a cut wound by barbed wire about 4 days ago. The skin surrounding the wound has become red, swollen and sore, and with pus. The patient confirmed that he had a recent tetanus vaccination booster.	No	0
	cloxacillin 250–500 mg 4 times daily, or dicloxacillin 250–500 mg 4 times daily	1

^1^ Antibiotics may be likely to ne of benefit for the patient. Consider no antibiotic with advice or an antibiotic treatment is based on pharmacist discretion.

## Data Availability

The data presented in this study are available on request from the corresponding author.

## Supplementary Materials

The following are available online at https://www.mdpi.com/2079-6382/10/2/154/s1. Supplemental Table S1: Selected provinces and number of selected pharmacies in each province.
