# Supplementary material for: Evaluation of Antibiotic Supply Decisions by Community Pharmacists in Thailand: A Vignette Study
Source: Antibiotics (Basel). 2021 Feb 3;10(2):154. doi: 10.3390/antibiotics10020154 (PMC7913721; doi:10.3390/antibiotics10020154)
Supplement: Supplementary file 1 [file antibiotics-10-00154-s001.pdf]

Table S1. Selected provinces and number of selected pharmacies in each province.

Regions	Population Sizes	Provinces	Number of Non-Accredited Pharmacies	Number of Selected Non-Accredited Pharmacies	Number of Accredited Pharmacies	Number of Selected Accredited Pharmacies	Number of Respondents (response rate)	Response Rate by Region
Central Thailand	> 1,000,000	Bangkok<br>Samut Prakan<br>NonThaburi	4870<br>524<br>631	205<br>22<br>27	363<br>40<br>51	128<br>15<br>19	70<br>(16.8%)	17.9%
	500,000–1,000,000	Nakhon Pathom<br>Chachoengsao<br>Samut Sakhon	311<br>164<br>273	13<br>10<br>11	19<br>8<br>8	10<br>8<br>8	15<br>(25.0%)	
	< 500,000	Phetchaburi<br>Nakhon Nayok<br>Trat	13<br>50<br>84	10<br>10<br>10	6<br>2<br>0	6<br>2<br>0	7<br>(18.4%)	
Northern Thailand	> 1,000,000	Chiang Mai<br>Chiang Rai<br>Nakhon Sawan	600<br>201<br>138	25<br>10<br>10	49<br>8<br>10	13<br>8<br>10	19<br>(25.0%)	25.2%
	500,000–1,000,000	Kamphaeng Phet<br>Sukhothai<br>Phichit	79<br>76<br>61	10<br>10<br>10	4<br>1<br>7	4<br>1<br>7	10<br>(23.8%)	
	< 500,000	Nan<br>Uttaradit<br>Uthai Thani	62<br>50<br>32	10<br>10<br>10	2<br>3<br>2	2<br>3<br>2	10<br>(27.0%)	
Northeastern Thailand	> 1,000,000	Nakhon Ratchasima<br>Udon Thani<br>Surin	323<br>290<br>133	14<br>12<br>10	20<br>8<br>5	10<br>8<br>5	15<br>(25.4%)	22.4%
	500,000–1,000,000	Kalasin<br>Maha Sarakham<br>Yasothon	134<br>157<br>72	10<br>10<br>10	2<br>8<br>2	2<br>8<br>2	10<br>(23.8%)	
	< 500,000	Bungkan<br>Amnat Charoen<br>Mukdahan	48<br>36<br>49	10<br>10<br>10	0<br>1<br>2	0<br>1<br>2	5<br>(15.2%)	
Southern Thailand	> 1,000,000	Nakhon Sithammarat<br>Songkhla<br>Surat Thani	262<br>376<br>651	10<br>15<br>26	7<br>43<br>27	7<br>16<br>10	20<br>(23.8%)	20.5%
	500,000–1,000,000	Narathiwat<br>Yala<br>Chumphon	72<br>59<br>86	10<br>10<br>10	6<br>6<br>2	6<br>6<br>2	8<br>(18.2%)	
	< 500,000	Phuket<br>Satun<br>Ranong	705<br>59<br>38	30<br>10<br>10	35<br>4<br>1	12<br>4<br>1	12<br>(17.9%)	
		**Missing**					7	
	**Total**		11,769	652	761	348	208<br>(20.8))	

To classify strata, firstly, community pharmacies were grouped based on their location into the four regions, which are central Thailand, Northern Thailand, North-eastern Thailand, and Southern Thailand. Secondly, community pharmacies in each regional area were divided into three groups based on the population size of the province in which the pharmacy was located. Three provinces in each of the three group size categories were randomly selected using a random number generator website,

https://stattrek.com/statistics/random-number-generator.aspx. This gave thirty-six provinces across four regional areas, covering three population levels.

As of May 2019, there were a small number of accredited pharmacies (The Office of Pharmacy Accreditation (Thailand) 2019), that is, 1054 out of 18,900 Type I community pharmacies (5.6%). Therefore, accredited community pharmacies in each province were selected by quota sampling in order to recruit a reasonable number of accredited pharmacies to the study (as one research question relates to comparing views of pharmacists working in accredited pharmacies versus those who did not). A ratio of approximately 1 to 2 accredited pharmacies to non-accredited pharmacies was used, resulting in approximately 350 accredited pharmacies and 650 non-accredited pharmacies in the final sample.

As there is a wide range in the number of community pharmacies in the different provinces, 35% of accredited pharmacies in each of the selected provinces were invited to participate in the study. Many provinces had few accredited pharmacies, so in cases where there were less than 10 accredited pharmacies in a selected province, all accredited pharmacies were invited. To recruit from the 650 non-accredited pharmacies, approximately 4% of non-accredited pharmacies were randomly selected, with at least 10 non-accredited pharmacies invited from each province. The list of provinces and the number of pharmacies eligible to participate in the study is shown in Table S1. From the resulting list of community pharmacies by province in each of the four regional areas, community pharmacies were randomly selected using a random number generator website, https://stattrek.com/statistics/random-number-generator.aspx.
